# Practice patterns and outcomes in the management of Thai patients with Graves’ disease

**DOI:** 10.1186/s13044-021-00097-y

**Published:** 2021-03-03

**Authors:** Yotsapon Thewjitcharoen, Krittadhee Karndumri, Waralee Chatchomchuan, Sriurai Porramatikul, Sirinate Krittiyawong, Ekgaluck Wanathayanoroj, Nampetch Lekpittaya, Worawit Kittipoom, Tawee Anuntakulnatee, Somboon Vongterapak, Siriwan Butadej, Soontaree Nakasatien, Rajata Rajatanavin, Thep Himathongkam

**Affiliations:** Diabetes and Thyroid Center, Theptarin Hospital, Bangkok, Thailand

**Keywords:** Graves’ disease, Practice patterns, Outcomes, Thai

## Abstract

**Background:**

The treatment of hyperthyroid Graves’ disease (GD) varies considerably among geographic areas. In this study, we aimed to evaluate practice patterns and treatment outcomes in Thai patients with hyperthyroid GD.

**Methods:**

A retrospective cohort study over 35 years (1985–2019) in patients with hyperthyroid GD was conducted. The trends of treatment options were compared periodically during the study period and the overall remission rate from each option was determined.

**Results:**

A total of 2736 hyperthyroid GD patients were treated and followed-up for at least 3 months over the study period (female 82.0%, mean age at diagnosis 36.3 ± 12.0 years, median duration of follow-up 74.5 months). Anti-thyroid drug (ATD) was the most commonly used treatment (78.0%), followed by RAI (21.0%), and surgery (1.0%). There was a significant downward trend for surgery, from 12.3% in the 1980s to only 0.2% in last phase of the study period. The preference for RAI therapy has also decreased in the last 5 years. Among ATD-treated patients, the remission rate was achieved only in 30.7 and 16.0% of all ATD-treated patients were eventually treated with RAI. Spontaneous hypothyroidism developed in 2.7% of the ATD-treated patients during a follow-up period. Almost all RAI-treated patients (97.1%) developed hypothyroidism.

**Conclusions:**

Our present study highlighted the changing landscape of primary treatments for hyperthyroid GD toward ATD and the sharp downward trend in the surgical option. Even though ATD was associated with a low remission rate, it was preferred by many patients and physicians. The use of RAI as the primary treatment decreased in the last decade. However, RAI was a very effective treatment for Graves’ hyperthyroidism but will inevitably induce hypothyroidism and a requirement for life-long replacement therapy.

**Supplementary Information:**

The online version contains supplementary material available at 10.1186/s13044-021-00097-y.

## Background

Hyperthyroid Graves’ disease (GD) is the most common cause of hyperthyroidism [1]. Despite radioactive iodine (RAI) being the most common recommended first-line treatment in the United States (U.S.) [2] and recently in the National Institute for Health and Care Excellence (NICE) guideline from United Kingdom [3] due to its effectiveness and prevention of relapse, the first-choice treatments for this disease varies among different countries [4–6]. While RAI is the preferred choice of treatment for practicing clinicians in the U.S., other parts of the world especially in Asia are likely to offer the anti-thyroid drug (ATD) as the initial option [7]. Choice of treatment requires physicians to discuss both long-term effectiveness and potential risks carefully with the patient. Even though the long-term follow-up on quality of life following treatment of GD showed similar efficacy among these three treatment options, each modality of treatment for GD has advantages and limitations [8]. Therefore, patients’ preferences after understanding all available treatment options would represent the reason for the selected choice of treatment.

In recent years, there has been growing concerns about the potential increased risk of malignancies following RAI and its risk for Graves’ ophthalmopathy (GO) [9, 10]. However, the remission rate from ATD is highly variable in most patients [11, 12]. According to the current American Thyroid Association (ATA) guideline, experts strongly recommended using definitive therapy in patients with high risk of disease recurrence [2]. For RAI therapy, since many methods for dose calculation have not been successful in guaranteeing long-term euthyroid status, many institutes now use fixed doses of RAI based on thyroid size and severity of thyroid status to ensure hypothyroidism as a goal for treatment. The role of thyroidectomy for GD patients as the first-line treatment is very limited at present (only less than 2–3% in most studies) but this treatment is still the most effective way to control hyperthyroidism immediately or in countries where RAI treatment is not readily available [13].

It is intriguing that GD treatment by thyroidologists had been changing toward ATD in recent years. To better understand the changing landscape of GD in Thai patients, this study aimed to evaluate clinical characteristics, practice patterns, and treatment outcomes in Thai patients with hyperthyroid GD.

## Methods

### Data sources

A retrospective cohort study over 35 years (1985–2019) in adult Thai patients with hyperthyroid GD seen at Theptarin Hospital, a private tertiary endocrine center in Bangkok was conducted. At our hospital, patients were self-referred and the cost of treatments had to be covered by their private health insurance or self-funded. Patients with other ethnicities, age < 15 years, GD in remission or GD with subclinical hyperthyroidism at the time of their initial visit, follow-up period < 3 months were excluded. All medical records were reviewed and analyzed with respect to their clinical characteristics, selected treatment modality, and the outcomes and recurrence of disease after each treatment course.

### Definitions and variables

The diagnosis of hyperthyroid GD was based on typical symptoms and signs such as a diffuse goiter, the presence of GO or dermopathy and biochemical evidence of overt hyperthyroidism and the presence of thyroid autoantibodies (TSH Receptor antibody;TRAb, anti-thyroid peroxidase antibody;anti-TPO, anti-thyroglobulin;anti-Tg). Newly diagnosed patients were defined as those with an onset of less than 3 months. Thyroid palpation data determined by treating physicians was transformed into thyroid volume on the basis of goiter size compared with normal thyroid size as following: small (barely palpable or ≤ 30 g), medium (2–3 times when compared with normal thyroid gland), and huge (more than 3 times or ≥ 60 g). Serum levels of thyroid stimulating hormone (TSH), free thyroxine (FT4), and total triiodothyronine (T3) were retrieved from the medical records of patients recently diagnosed with GD and treated with ATD. Serum T3, FT4, and TSH concentrations were measured by electrochemiluminescent immunoassays (Roche Diagnostics, Indianapolis, USA). The reference ranges used for serum T3, FT4 and TSH levels were 60–177 ng/dL, 0.9–1.7 ng/dL, and 0.3–4.2 mIU/L, respectively. Anti-TPO and anti-Tg were measured using electrochemiluminescent immunoassay (Roche Diagnostics, Indianapolis, USA). Normal values were defined as follows: Anti-TPO < 34 IU/mL and Anti-Tg < 115 IU/mL.

During the study period, 15 thyroidologists treated the patients. The choice of primary treatment was chosen based on patient preference and shared decision making which did not change throughout the study period. In patients selecting ATD as their treatment option, our routine practice was to administer methimazole (MMI) or propylthiouracil (PTU) for at least 12–18 months. Remission of GD was defined as patients with normalized serum thyroid stimulating hormone (TSH) without ATD for at least 12 months [2].

If RAI was selected, a single fixed dose of RAI based on estimated thyroid size was prescribed individually by the respective thyroidologist. Cure for hyperthyroidism after RAI was defined as having euthyroid status for 6 months without any treatment or the need for levothyroxine replacement for post-treatment hypothyroidism. Generally, our practice in using RAI treatment was to administer one dose of RAI aiming to achieve hypothyroidism. Treatments failure from RAI or surgery was defined if patients required subsequent treatments (ATD, another dose of RAI or another operation) after 6 months following their initial treatment. Final disease status was determined based on the last clinical visit during study period. This study was approved by the Institutional Review Board committee of Theptarin Hospital (EC No.4–2019).

### Statistical analysis

Descriptive statistical analysis was used for baseline characteristics and result for each treatment modality. Data was reported in mean with standard deviation, median with interquartile range, and number with percentage. Differences in the mean or median between groups were analyzed using a t test and ANOVA test. To assess the trend of practice patterns and outcomes, the 35-year study period was divided into 3 intervals of 10 years and the last interval of 5 years as following: 1985–1994, 1995–2004, 2005–2014, and 2015–2019 for comparison. In newly diagnosed GD patients treated with ATD, patients who achieved remission and those who did not were compared with t- test for continuous data and Chi-square for categorical data. Skewed distribution continuous variables were compared by Mann-Whitney U test. Serum T3 and FT4 values were transformed into the quartile category and the highest quartile was compared with the remaining quartiles for sustained disease remission. Variables with established association with disease remission were selected for univariate analysis, and those with a *P*-value < 0.1 were included in the multivariate logistic regression analysis to determine associated clinical factors and disease remission. *P* <  0.05 was considered statistically significant. All statistical analyses were performed using the SPSS Statistical Package, version 20 (IBM Corp., Armonk, NY, USA).

## Results

### Patient characteristics

**Fig. 1:**
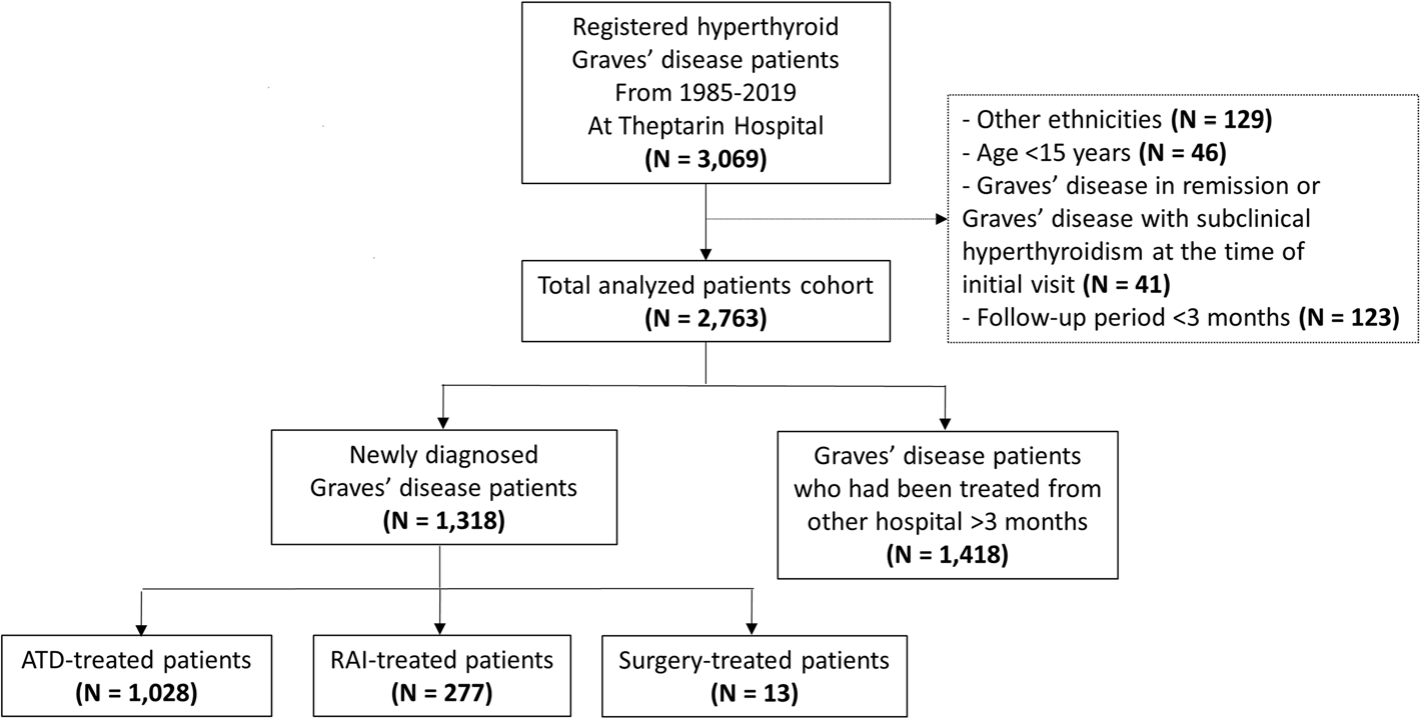
Flowchart illustrating the selected patients in the study

A total of 3069 adult Thai patients with hyperthyroid GD were registered at Theptarin hospital from 1985 to 2019. Of those, 2736 patients (89.1%) who met the criteria were included in this study as shown in Fig. 1. The baseline demographic data and results of thyroid serum autoantibodies were demonstrated in Table 1. In this overall cohort, the mean age at diagnosis was 36.3 ± 12.0 years and median duration of follow-up 74.5 months (IQR 36.0–143.5 months). Family history of thyroid disorders was found in 41.2%. The presence of moderate-to-severe GO was 2.8% and only 0.1% of patients had pretibial myxedema. Thyrotoxic periodic paralysis (TPP) was found in 62 patients or 2.3% of all patients (male 95.2%, age at presentation 35.7 ± 8.2 years). TPP was the first presentation of hyperthyroid GD in 82.3% of these patients. Of the 2736 patients, 1318 patients (48.2%) were newly diagnosed GD patients. The details of newly diagnosed GD patients in each interval period were shown in Supplementary Table 1.

**Table 1:** Clinical characteristics and results of thyroid autoantibodies stratified by interval period over 35 years

	Total (*N* = 2736)	1985–1994 (*N* = 144)	1995–2004 (*N* = 416)	2005–2014 (*N* = 1209)	2015–2019 (*N* = 967)
Female (%)	82.0%	87.5%	85.6%	80.3%	81.7%
Age at diagnosis	36.3 + 12.0	33.0 + 9.7	34.7 + 10.9	36.0 + 12.4	37.8 + 12.1
- Age < 40 years	80.5%	72.2%	70.2%	66.9%	62.0%
- Age 40–59 years	35.8%	27.8%	28.6%	27.5%	32.3%
- Age ≥ 60 years	5.7%	–	1.2%	5.6%	5.7%
Family history of thyroid disorders (%)	41.2%	30.6%	38.0%	44.3%	40.1%
Smoking status (%)					
-Non-smoker	96.4%	97.9%	98.0%	96.7%	95.1%
-Ex-smoker	0.9%	–	1.0%	0.7%	1.3%
-Active smoker	2.7%	2.1%	1.0%	2.6%	3.5%
Weight status at initial presentation					
-Weight loss	85.0%	81.2%	83.9%	84.4%	86.8%
-Weight neutral	11.6%	13.2%	13.0%	12.6%	9.5%
-Weight gain	3.4%	5.6%	3.1%	3.0%	3.7%
Estimated thyroid size					
-Small	43.0%	25.0%	41.8%	46.4%	41.9%
-Medium	41.5%	54.9%	42.6%	38.6%	42.6%
-Huge	15.5%	20.1%	15.6%	15.0%	15.5%
Graves’ Ophthalmopathy(%)					
-No GO	83.9%	77.8%	79.3%	87.8%	81.9%
-Mild GO	13.3%	21.5%	19.2%	9.6%	14.1%
-Moderate to Severe GO	2.8%	0.7%	1.5%	2.6%	4.0%
Pretibial myxedema	0.1%	–	–	0.1%	0.2%
Thyrotoxic periodic paralysis (%)	2.3%	–	3.1%	1.8%	2.8%
Coexisting thyroid disease					
- Solitary thyroid nodule	2.2%	3.5%	1.7%	2.3%	2.1%
`- Multinodular goiter	3.4%	2.1%	1.4%	3.4%	4.3%
- Thyroid cancer	0.2%	–	0.2%	0.2%	0.3%
Positive Anti-TPO (%)^a^	71.9%	73.8%	77.6%	75.8%	65.6%
Positive Anti-Tg (%)^b^	61.3%	31.7%	53.6%	71.7%	52.4%

^a^Available data 2071/2736 ^b^Available data 2023/2736

Based on available results of serum thyroid autoantibodies, the rate of antibody positivity was 71.9% for anti-TPO and 61.3% for anti-Tg. Both anti-TPO and anti-Tg were found in 52.3% of the patients. One-fifth of the patients were negative for both antibodies. Of 88.1% of those patients who were tested negative for anti-TPO and anti-Tg were positive for TRAb. However, serum TRAb was measured at the initial visit only in 3.7% of patients.

### Practice patterns and outcomes of treatments

**Fig. 2:**
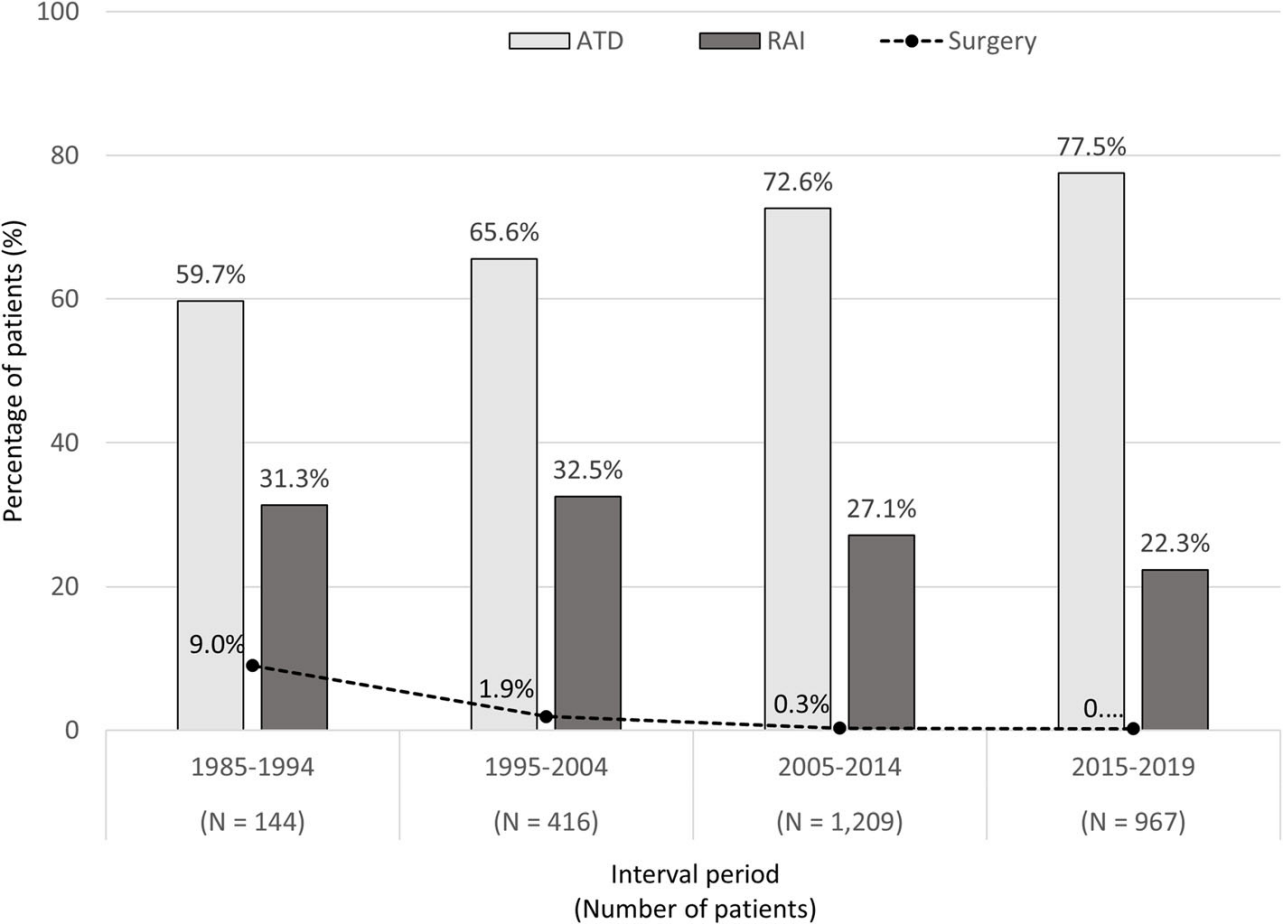
Trends of each treatment modality in a total cohort stratified by each interval period over 35 years

Among all patients, ATD was the most preferred choice (72.6%), followed by RAI treatment (26.5%), and surgery (0.9%). The MMI was chosen in 92.2% of ATD-treated patients. Block and replacement regimen (a combination of ATD and levothyroxine) was used in only 1.9% of patients. In RAI-treated patients, a median dose of 15 mCi (range 5–30 mCi) was given. Of these, 30.7% of RAI treatments were administered due to relapsed Graves’ disease after ATD withdrawal. Almost all RAI-treated patients (97.1%) developed hypothyroidism. Surgical treatment was performed as total thyroidectomy in 57.7% (15/26) and subtotal thyroidectomy in the remaining patients. Relapsed GD after surgery was noted in 4 patients (15.4%) (all of them underwent subtotal thyroidectomy) which 2 patients later received RAI treatment as another ablative treatment. The trends of each treatment modality stratified by each interval period were demonstrated in Fig. 2.

**Fig. 3:**
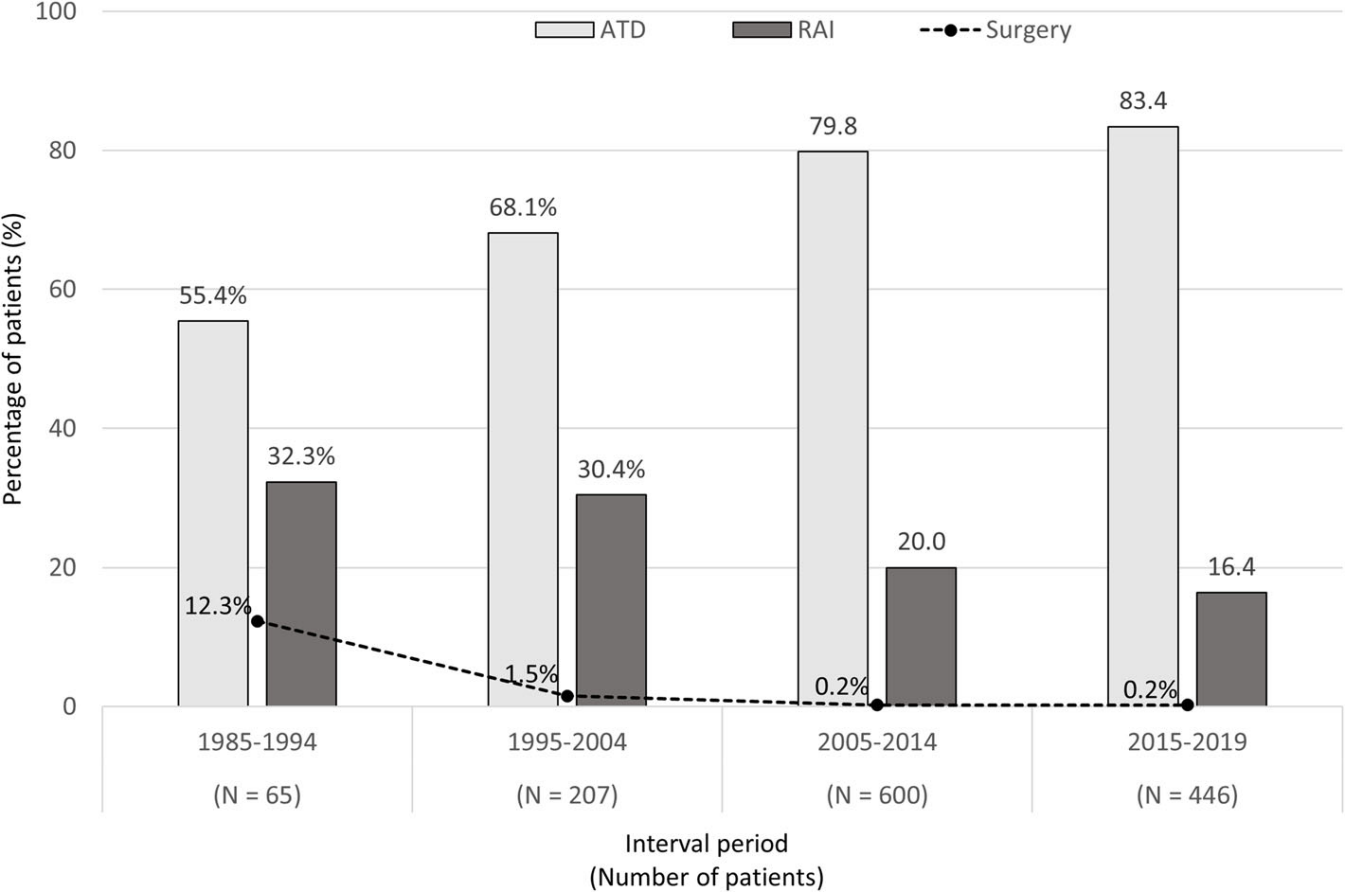
Trends of each treatment modality in a cohort of newly diagnosed Graves’ disease patients stratified by each interval period over 35 years

When the cohort of newly diagnosed GD patients was analyzed (*N* = 1318), a shift was observed toward opting ATD as a primary treatment (increased from 55.3 to 83.4%, *p*-value < 0.001) and sharply decreased in surgery (decreased from 12.3 to 0.2%, p-value < 0.001). The trend for RAI therapy has also decreased in the last 5 years period as shown in Fig. 3. Among ATD-treated patients, the remission rate was achieved in only 30.7% of patients at the time of their last clinical evaluation. The median follow-up period after ATD withdrawal was 36 months (IQR 12–84 months). During the follow-up period, 16.0% of all ATD-treated patients were eventually treated with RAI. Relapse after completion of ATD treatment regimen was noted in 178 patients (56.3%). In only 25 cases of these patients (14.0%) could withdraw ATD after having received the second course of ATD treatment. Spontaneous hypothyroidism developed in 2.3% of ATD-treated patients during the follow-up period. A comparison between ATD-treated patients who could achieve disease remission and patients who could not was shown in Table 2. Multivariate analysis revealed that patients younger than 40 years at diagnosis, the presence of GO, and the high initial serum T3 were independently associated with non-sustainable remission as demonstrated in Table 3.

**Table 2:** Comparison between ATD-treated patients who could achieve disease remission and patients who could not achieve remission in newly diagnosed Graves’ disease patients

	Patients who achieved remission (*N* = 316)	Patients who could not achieve remission (*N* = 712)	*p-value*
Female (%)	260 (82.3%)	583 (81.9%)	0.930
Duration of follow-up (months)	89.1 + 70.6	95.5+ 85.9	0.209
Age at diagnosis	38.2 + 11.9	35.8 + 12.1	0.007
- Age < 40 years	188 (59.5%)	488 (68.5%)	
- Age 40–59 years	111 (35.1%)	182 (25.6%)	
- Age ≥ 60 years	17 (5.4%)	42 (5.9%)	
Estimated thyroid size			0.001
-Small	190 (60.1%)	358 (50.3%)	
-Medium	114 (36.1%)	287 (40.3%)	
-Huge	12 (3.8%)	67 (9.4%)	
Active smoker	3 (0.9%)	18 (2.5%)	0.149
Presence of GO	24 (7.6%)	87 (12.2%)	0.029
TSH (mIU/L)	0.015 + 0.044	0.011 + 0.017	0.237
FT4 (ng/dL)	4.03 + 1.88	4.54 + 1.92	0.001
T3 (ng/dL)	340.54 + 145.86	408.32 + 167.03	< 0.001

**Table 3:** Univariate and multivariate analysis of clinical factors and laboratory data for predicting sustained remission after completion of standard course of ATD

	Univariate analysis			Multivariate analysis		
	HR	CI	*p-value*	HR	CI	*p-value*
Female (%)	0.97	0.69–1.38	0.879			
Age at diagnosis - Age < 40 years	0.67	0.51–0.89	0.005	0.75	0.56–0.99	0.049
Estimated thyroid size - medium to huge	0.67	0.51–0.88	0.004	0.79	0.59–1.06	0.114
Active smoker	0.37	0.11–1.26	0.113			
Presence of GO	0.59	0.37–0.95	0.029	0.59	0.37–0.95	0.030
High FT4 (4th Quartile)	0.64	0.45–0.93	0.018	0.87	0.58–1.29	0.486
High T3 (4th Quartile)	0.49	0.31–0.76	0.001	0.58	0.36–0.95	0.029

### Adverse events from various treatment choices

Adverse events were rare with only one case diagnosed with agranulocytosis in ATD-treated patient. In RAI-treated patients, there were four cases with new-onset or worsening GO after RAI. The rate of permanent hypoparathyroidism after surgery was found only in 1 case of all surgery-treated patients and there was no report of permanent recurrent laryngeal nerve injury in this cohort.

## Discussion

This study was the first to comprehensively analyze the practice patterns and outcomes in the management of hyperthyroid GD in Southeast Asians over three decades. As in other Asian studies, ATD as the first-line treatment has constantly increased over time in Thailand. Among ATD-treated newly diagnosed patients, the remission rate after the standard course of ATD at least 12–18 month was in line with previous studies [14, 15] over a median duration of follow-up period at 6.2 years. Remission rate from ATD as a primary treatment is difficult to predict in routine clinical practice. Even though major guidelines suggested definite RAI therapy as a primary treatment option for most patients [2, 3], there was a declining trend in RAI over a 35-year period at our hospital.

Regardless of the chosen treatment method for hyperthyroid GD, a recent study from the United Kingdom demonstrated that early and intensive control of hyperthyroidism improved long-term cardiovascular morbidity and all-cause mortality [16]. To identify the best treatment for each patient, clinicians need to engage their patients into a shared decision-making process based on patients’ co-morbidities, personal expectations and value. Several risk factors predicting relapse after completing the course of ATD have been reported, e.g. younger age, large goiter, smokers, male sex, and higher levels of serum TRAb before ATD discontinuation [17–19]. However, the risk of relapse is still difficult to predict in the long-term even in patients who do not have all suggested risk factors for relapse. After the introduction of iodine supplementation in Thailand over the past 3 decades, most parts of Thailand especially Bangkok are iodine-sufficient area [20]. The increase in iodine intake may adversely affect the remission rate in ATD treated patients [21]. When compared our current study with a previous study among Thai patients with GD since 1970s to determine the remission rate from ATD [22], our current cohort revealed a lower remission rate (30.7% versus 50.0%). Moreover, as revealed in our present study and others, spontaneous hypothyroidism after ATD withdrawal could develop in up to 2–5% of ATD-treated patients [12]. Therefore, lifelong follow-up is required in all patients with GD.

The choice of treatment for GD varied widely over many countries; however, ATD was preferred by nearly all patients in Asian countries [5–8]. Our study showed the rate of RAI as choice of treatment in around one-fifth of newly diagnosed patients which was in between the United State and European countries and was higher according to a recent online survey among Thai endocrinologists where ATD was the preferred choice in more than 90% of all respondents [6]. Although RAI has been considered a safe and effective treatment for GD for more than seven decades, growing concerns remain regarding the risk of malignancy and impaired quality of life [23]. These issues might have influenced the decision-making process for some patients. However, it should be emphasized that ablative treatment rather than continuing long-term ATD therapy might be the more appropriate choice for some patients.

Consistent with other reports, the trend of surgery as another option for definitive treatment for GD has decreased sharply according to our study. In the past, subtotal thyroidectomy was the preferred choice to minimize post-surgical complications and postoperative hypothyroidism. However, it became clear that up to 10% of patients who underwent this approach experienced relapsed GD from ongoing autoimmune processes [24]. Currently, total or near-total thyroidectomy is the standard procedure to treat GD in our center by a highly experienced thyroid surgeon. Even though the role of surgery as a primary treatment for GD is limited, this treatment option is still a viable option for patients who need to control their hyperthyroidism rapidly, patients who have huge goiters, or patients with suspected co-existing thyroid cancer.

There were several limitations which could have influenced our results. First, the inherent weakness of the retrospective study and data source from only single center should be acknowledged. Health system in Thailand is composed of universal healthcare coverage, social health insurance, civil servant medical benefit system, and self-payment [25]. In our center which is a private hospital, patients have to pay on themselves or use their personal insurance. The cost of different treatment modalities varied considerably and depends on the reimbursement scheme. For the cost of treatments at our private center, the choice of surgery is about ten times more expensive when compared with the RAI treatment. Therefore, financial issue could potentially affect type of patients who visit our hospital and type of chosen treatment. Further studies in nationwide survey should be conducted to verify the changing trends of treatment among Thai patients with GD. Second, serum TRAb was introduced in our center from 2010 but used infrequently for establishing the diagnosis of GD from its high cost and long turnaround time. Radioiodine uptake was also done infrequently. However, the clinical features of GD (including family history, the presence of other autoimmune diseases, ophthalmopathy, and dermopathy) and clinical course of the disease made the chance for misdiagnosis quite low. Third, there were many relevant missing data in medical records such as complete biochemical data, objective measurements of the thyroid gland size by ultrasound, estimation and GO assessment, documented reasons for selected choice of treatments, etc. However, our present data is one of the largest cohort studies with longitudinal data of Asian patients over 3 decades. Our study can give an insight into the changing trends of thyroidologists’ treatment preferences in Thai patients with GD. Objective tools to document the process of shared decision-making approach in counseling and selecting the treatment of choice for GD should be adopted as previously reported in a recent study [26]. Second, some important parameters to predict the remission rate from ATD had been omitted from logistic regression analysis due to very few available data such as serum free triiodothyronine (*FT3*), levels of thyroid autoantibodies, initial serum TRAb levels, etc. Third, the ultimate long-term outcomes from GD treatments including quality of life and mortality could not be assessed.

## Conclusion

In conclusion, our present study highlights the changing landscape of treatment of choice for hyperthyroid GD toward ATD and decreased RAI and in the surgical option. Remission rates from ATD were low and consistent with previous studies. RAI is a very effective treatment for Graves’ hyperthyroidism but will inevitably induce hypothyroidism and a requirement for life-long replacement therapy.

## Supplementary Information

**Additional file 1: Supplement Table 1.** Clinical characteristics of newly diagnosed Graves’ disease patients stratified by interval period over 35 years.

## Data Availability

The dataset supporting the conclusions of this article is available on request.

## Abbreviations

ATA: American Thyroid Association
ATD: Antithyroid drug
GD: Graves’ disease
GO: Graves’ ophthalmopathy
NICE: National Institute for Health and Care Excellence
RAI: Radioactive iodine
T3: Total triiodothyronine
FT4: Free thyroxine
TSH: Thyroid stimulating hormone
anti-TPO: Anti-thyroid peroxidase antibody
anti-Tg: Anti-thyroglobulin
PTU: Propylthiouracil
MMI: Methimazole
TPP: Thyrotoxic Periodic Paralysis
TRAb: Thyroid stimulating hormone receptor autoantibody
