# Supplementary material for: Practice patterns and outcomes in the management of Thai patients with Graves’ disease
Source: Thyroid Res. 2021 Mar 3;14:5. doi: 10.1186/s13044-021-00097-y (PMC7927256; doi:10.1186/s13044-021-00097-y)
Supplement: Supplementary file 1 — Additional file 1: Supplement Table 1. Clinical characteristics of newly diagnosed Graves’ disease patients stratified by interval period over 35 years. [file 13044_2021_97_MOESM1_ESM.docx]

**Supplementary data**

**Supplement Table 1**. Clinical characteristics of newly diagnosed Graves’ disease patients stratified by interval period over 35 years

|  | Total  (N= 1,318) | 1985-1994  (N= 65) | 1995-2004  (N= 207) | | 2005-2014  (N= 600) | | 2015-2019  (N= 446) | |
| --- | --- | --- | --- | --- | --- | --- | --- | --- |
| Female (%) | 82.0% | 90.8% | | 87.4% | | 80.2% | | 80.7% |
| Age at diagnosis  - Age < 40 years  - Age 40-59 years  - Age≥ 60 years | 36.4+11.9  66.1%  28.8%  5.1% | 33.1+8.8  75.3%  24.7%  - | | 34.9+10.9  68.6%  30.4%  1.0% | | 36.5+12.2  65.2%  29.0%  5.8% | | 37.6+12.1  64.8%  28.5%  9.7% |
| Family history of thyroid disorders (%) | 42.4% | 26.1% | | 36.2% | | 45.3% | | 43.7% |
| Smoking status (%)  -Non-smoker  -Ex-smoker  -Active smoker | 97.3%  0.6%  2.1% | 100.0%  -  - | | 99.5%  -  0.5% | | 97.0%  0.5%  2.5% | | 96.2%  1.1%  2.7% |
| Weight status at initial presentation  -Weight loss  -Weight neutral  -Weight gain | 83.5%  12.6%  3.9% | 81.6%  9.2%  9.2% | | 83.1%  13.0%  3.9% | | 83.3%  14.0%  2.7% | | 84.1%  11.0%  4.9% |
| Estimated thyroid size  -Small  -Medium  -Huge | 46.1%  42.7%  11.2% | 18.5%  63.0%  18.5% | | 44.0%  45.4%  10.6% | | 51.8%  38.0%  10.2% | | 43.5%  44.8%  11.7% |
| Graves’ Ophthalmopathy(%)  -No GO  -Mild GO  -Moderate to Severe GO | 88.5%  9.9%  1.6% | 81.5%  16.9%  1.6% | | 83.6%  16.4%  - | | 91.7%  6.8%  1.5% | | 87.7%  10.1%  2.2% |
| Pretibial myxedema | 0.2% | - | | - | | - | | 0.5% |
| Thyrotoxic periodic paralysis (%) | 2.4% | - | | 3.4% | | 1.8% | | 2.9% |
| Coexisting thyroid disease  - Solitary thyroid nodule  - Multinodular goiter  - Thyroid cancer | 2.4%  3.0%  0.2% | 4.6%  3.1%  - | | 2.9%  0.5%  0.5% | | 2.3%  3.2%  0.2% | | 1.8%  4.0%  0.2% |
| Positive Anti-TPO (%)* | 68.5% | 77.8% | | 74.1% | | 72.4% | | 60.8% |
| Positive Anti-Tg (%)^#^ | 64.1% | 59.3% | | 48.2% | | 72.4% | | 58.8% |

*Available data 993/1,318 ^#^Available data 964/1,318
